# Diversity and Activity of Sulfate-Reducing Prokaryotes in Kamchatka Hot Springs

**DOI:** 10.3390/microorganisms9102072

**Published:** 2021-10-01

**Authors:** Evgenii N. Frolov, Alexandra V. Gololobova, Alexandra A. Klyukina, Elizaveta A. Bonch-Osmolovskaya, Nikolay V. Pimenov, Nikolay A. Chernyh, Alexander Y. Merkel

**Affiliations:** 1 Winogradsky Institute of Microbiology, Federal Research Center of Biotechnology of the Russian Academy of Sciences, 60 let Oktjabrja pr-t, 7, bld. 2, 117312 Moscow, Russia; sasha.gololobova@yandex.ru (A.V.G.); alexandra.a.popova@gmail.com (A.A.K.); elizaveta.bo@gmail.com (E.A.B.-O.); npimenov@mail.ru (N.V.P.); chernyh3@yandex.com (N.A.C.); alexandrmerkel@gmail.com (A.Y.M.); 2 Faculty of Biology, Lomonosov Moscow State University, 1-12 Leninskie Gory, 119991 Moscow, Russia

**Keywords:** Kamchatka Peninsula, terrestrial hot springs, microbial community, sulfate-reducing prokaryotes, *dsrB* profiling, 16S rRNA taxonomic profiling, radiotracing experiments, cultivation

## Abstract

Microbial communities of the Kamchatka Peninsula terrestrial hot springs were studied using radioisotopic and cultural approaches, as well as by the amplification and sequencing of *dsrB* and 16S rRNA genes fragments. Radioisotopic experiments with ^35^S-labeled sulfate showed that microbial communities of the Kamchatka hot springs are actively reducing sulfate. Both the cultivation experiments and the results of *dsrB* and 16S rRNA genes fragments analyses indicated the presence of microorganisms participating in the reductive part of the sulfur cycle. It was found that sulfate-reducing prokaryotes (SRP) belonging to *Desulfobacterota*, *Nitrospirota* and *Firmicutes* phyla inhabited neutral and slightly acidic hot springs, while bacteria of phylum *Thermodesulofobiota* preferred moderately acidic hot springs. In high-temperature acidic springs sulfate reduction was mediated by archaea of the phylum *Crenarchaeota*, chemoorganoheterotrophic representatives of genus *Vulcanisaeta* being the most probable candidates. The 16S rRNA taxonomic profiling showed that in most of the studied communities SRP was present only as a minor component. Only in one microbial community, the representatives of genus *Vulcanisaeta* comprised a significant group. Thus, in spite of comparatively low sulfate concentrations in terrestrial hot springs of the Kamchatka, phylogenetically and metabolically diverse groups of sulfate-reducing prokaryotes are operating there coupling carbon and sulfur cycles in these habitats.

## 1. Introduction

Dissimilatory sulfate reduction is a process of enormous environmental and biogeochemical relevance, which is carried out by numerous SRP. The vast variety of currently known SRP includes species belonging to four phylogenetic lineages of bacteria (*Desulfobacterota*, *Nitrospirota*, *Thermodesulfobiota* and *Firmicutes*), and two phyla of archaea (*Euryarchaeota* and *Crenarchaeota*) [1,2,3]. The *Desulfobacterota* phylum was recently proposed as a result of the reclassification of the proteobacterial classes *Deltaproteobacteria* and *Oligoflexia*, and the phylum *Thermodesulfobacteria* [3]. Currently, *Desulfobacterota* includes the taxa previously classified in the phylum *Thermodesulfobacteria*, and these reclassifications imply that the ability of sulfate reduction was vertically inherited in the *Thermodesulfobacteria* rather than laterally acquired as previously inferred [3]. The ability to perform sulfate reduction was predicted also for “*Candidatus Thermonerobacter thiotrophicus*”, which is affiliated with the *Bacteroides*/*Ignavibacteria*/*Chlorobi* group [4].

SRP inhabit diverse anaerobic environments that vary strongly in their physicochemical characteristics. Most SRP are mesophiles, but there are also several thermophilic species. Thermophilic SRP includes representatives of bacterial genera *Thermodesulfovibrio*, *Thermodesulfobacterium*, *Desulfovirgula*, *Desulfosoma*, *Thermodesulfobium*, *Thermodesulforhabdus*, *Ammonifex*, *Desulfotomaculum* and some of the hyperthermophilic archaea, such as *Archaeoglobus* and *Vulcanisaeta* [2,5]. SRP were isolated from terrestrial hot springs [6], deep-sea vents [7], bioreactors [8], high-temperature oil reservoirs and other geothermally heated subsurface environments [9,10]. The only genus containing both mesophilic and thermophilic species is the genus *Desulfotomaculum*; due to the ability of spore formation, thermophilic *Desulfotomaculum* were present in cold habitats [11]. Still, terrestrial hot springs are so far the most frequent sources of thermophilic SRP isolation. However, not much is known up to now about their distribution, relative abundance and activity in hot springs and hot pools of volcanic origin.

The Kamchatka Peninsula is one of the biggest areas of modern volcanic activity on the Earth, where more than a hundred volcanoes and numerous hot springs associated with geothermal activity are located. The systematic study of thermophilic microbial communities in Kamchatka started in the 1980s and usually was conducted in the Uzon Caldera. It is located in the South-Eastern part of Kamchatka and contains numerous hot springs and pools with temperatures from 40 to 96 °C [12,13,14]. During previous years, a large number of thermophilic prokaryotes belonging to different metabolic groups were isolated from Kamchatkan hot springs, among them only three species of thermophilic SRP: *Ammonifex thiophilus* [15], *Thermodesulfobium acidiphilum* [16] and *Desulfothermobacter acidiphilus* [17]. Based on genomic analysis, as well as on proteomic and cultural experiments, it was shown that “*Candidatus Vulcanisaeta moutnovskia*” isolated from a hot spring at the bottom of Mutnovskii volcano [18,19], was capable of sulfate reduction [5]. The activity of SRP was demonstrated for Termofilnyi Spring [20], Bourlyashchy Pool [21], Oreshek Spring and Oil Site [5,22] by in situ experiments with ^35^S-labelled sulfate. The 16S rRNA genes analysis, as well as metagenomic analyses, were performed for microbial communities of Kamchatka hot springs [13,14,23,24,25,26,27,28,29], but the diversity of SRP in Kamchatka hot springs was never discussed.

High-throughput sequencing of 16S rRNA gene fragments enables extensive alpha- and beta-diversity analyses of microbial communities. However, the detection of environmental SRP only by 16S rRNA gene-based methods is difficult because SRP belong to diverse and non-monophyletic lineages; they also are often related to non-SRP [30,31]. Instead, the dissimilatory (bi)sulfite reductase (*dsrAB*) genes-based methods were considered to be more adequate for this purpose [30,31,32,33]. *dsrAB* may be exploited as a phylogenetic marker in amplicon sequencing-based environmental studies [33,34,35,36,37,38,39,40,41]. Phylogenetic analysis discerns three main DsrAB protein families: reductive bacterial-type, reductive archaeal-type and oxidative bacterial-type DsrAB [32]. High-coverage PCR primer sets targeting reductive bacterial-type *dsrA* and *dsrB* genes and producing amplicon suitable for Illumina sequencing, as well as bioinformatics workflow for processing and taxonomic classification of short *dsrA* and *dsrB* reads were developed by Pelikan and colleagues [33].

In the present study, we used a primer set to match almost all known sequences of reductive bacterial-type *dsrB* [33], while the high-throughput sequencing of 16S rRNA gene fragments was used for the determination of the share of SRP present in studied microbial communities. As a result, we detected and investigated dissimilatory sulfate reduction in hot springs of Uzon Caldera, Mutnovskii Volcano and Geyser Valley with different physicochemical characteristics and also described the composition of corresponding microbial communities in order to define the diversity and distribution of SRP in terrestrial hydrothermal ecosystems of Kamchatka.

## 2. Materials and Methods

### 2.1. Sample Collection and Analytical Techniques

Samples of hot springs water and sediments were collected in 2015 at Central, West, East, and Orange Hydrothermal Fields of the Uzon Caldera, in Geyser Valley and at the bottom of Mutnovskii Volcano, Kamchatka. Sample of heated soil and groundwater was collected on Oil Site. Samples from thermal springs were collected into 50-mL glass vials with gas-tight butyl rubber stoppers and aluminum screw caps, which were completely filled, tightly closed, sealed, and transported to the laboratory at ambient temperature. For DNA isolation, samples of sediments were taken aseptically in 2-mL Eppendorf tubes with screw caps and then fixed with RNAlater™ Stabilization Solution (Thermo Fischer Scientific, Waltham, MA, USA). During transportation and storage, the fixed samples were maintained at +4 °C and then stored at −20 °C until the DNA was extracted. 

Sulfate ion concentrations were determined using Stayer ion chromatographer (Russia). Sulfide formation was determined using the colorimetric method with N,N-dimethyl-para-phenylenediamine as proposed by Trüper and Schlegel [42]; the developing blue coloration was subsequently quantified by spectrophotometry at λ = 670 nm.

### 2.2. Radiotracing Experiments

Sulfate reduction rates (SRR) were measured by means of the radioisotope technique using ^35^S-sulfate. Radioisotope experiments were performed in the laboratory 10‒14 days after sampling using 17-mL Hungate tubes with screw caps. Each tube contained a 6-mL specimen aliquot with 1:1 sediment to liquid phase ratio. The headspace was filled with 100% CO_2_, and 0.2 mL ^35^S-sulfate (Na_2_[^35^S]SO_4_, special activity 55.28 TBq/mmol, Perkin Elmer, MA, USA, 3μ Ci per sample) were injected into each tube with a syringe through the stopper. To assess the influence of energy substrates on sulfate reduction rate, six different substrates were tested: hydrogen (the head space was filled with H_2_/CO_2_ = 4/1), acetate, lactate, ethanol, methanol (10 mM each) and yeast extract (0.5 g/L). The tubes were incubated at temperatures corresponding to those of the sampling sites for 3 days; after incubation, the processes were stopped by the addition of 1 mL of 2 M NaOH. The subsequent treatment was performed as described previously [43].

### 2.3. DNA Extraction, 16S rRNA and dsrB Genes Amplification, Sequencing and Analyses

DNA from the samples was isolated as previously described [44]. Libraries of the V3–V4 region of the 16S rRNA gene and of the 1762–2107 region of the *dsrB* gene were prepared as previously described [45] with some modification. The following primer set was used for amplification of the V3–V4 region of the 16S rRNA gene: forward primer (5′-CAAGCAGAAGACGGCATACGAGATGTGACTGGAGTTCAGACGTGTGCTCTTCCG-ATCT XXXXXXXXXXXX ZZZZ CCTAYGGGDBGCWSCAG-3′) consisting of 5′ Illumina Linker Sequence, Index 1 (X), Heterogeneity Spacer [45] and 341F primer [46] respectively and reverse primer (5′-AATGATACGGCGACCACCGAGATCTACACTCTTTCCCTACACGACGCTCTTCCGATCT XXXXXXXXXXXX ZZZZ GACTACNVGGGTMTCTAATCC-3′) consisting of 3′ Illumina Linker Sequence, Index 2 (X), Heterogeneity Spacer (Z) and Pro-mod-805R primer [47], respectively. For amplification of the 1762–2107 region of the *dsrB* gene the same construction was used, but with DSR1762Fmix-DSR2107Rmix primer mix [33] on 3′-ends. PCR was conducted as previously described [14]. For each DNA sample, two libraries were prepared, which were sequenced in parallel using the MiSeq Reagent Micro Kit v2 (500-cycles) MS-103-1002 (Illumina, San Diego, CA, USA) on a MiSeq sequencer (Illumina, San Diego, CA, USA) according to the manufacturer’s recommendations. All the sequencing data are deposited in NCBI BioProject PRJNA753547.

### 2.4. Bioinformatics Processing and Data Analyses

Removal of adapters and length- and quality-based trimming was carried out using Cutadapt [48] and Trimmomatic [49]. Demultiplexing was carried out using deML [50]. Amplicon sequence variant (ASV) table was produced using Dadaist2 [51]. The 16S rRNA gene ASVs were classified according to Silva 138.1 taxonomic database [52]. *dsrB* gene sequences ASVs were classified based on a phylogenetic tree that was constructed by using database from Pelikan et al. [33] and IQ-TREE 2 software [53]. Visualization of data was partly performed in an R software using ggplot2 package [54].

### 2.5. Cultivation and Identification of SRP

Enrichment cultures were initiated by 10% (*w*/*v*) inoculation of anaerobically prepared, bicarbonate-buffered, sterile (by autoclaving at 121 °C for 1 h) liquid medium of the following composition (gram per liter distilled water, g L^−1^): NH_4_Cl, 0.33; KCl, 0.33; MgCl_2_·2H_2_O, 0.33; CaCl_2_·6H_2_O, 0.33; KH_2_PO_4_, 0.33; Na_2_SO_4_, 2.0; trace element solution [55], 1 ml; vitamin solution [56], 1 ml. Sodium sulfide (0.5 g L^−1^) was used as a reducing agent. Resazurin (1.0 mg L^−1^) was added as a redox indicator. Yeast extract (1 g L^−1^), acetate (10 mM), lactate (10 mM), ethanol (20 mM), methanol (20 mM), or H_2_ as a mixture with CO_2_ (4/1) were used as growth substrates. To adjust the pH of the medium to 4.5, 5.0, 5.5, 6.0 or 6.5, 3 N HCl and 3 N NaOH were used. The medium was dispensed in 5 mL aliquots into 17 mL Hungate tubes; the head space, if not indicated additionally, was filled with CO_2_. Pure cultures were obtained after multiple serial dilutions to extinction on the same medium. The 16S rRNA genes of new isolates were amplified and sequenced as described previously [57]. The taxonomic position of the isolates was determined by comparing the almost complete sequences of their 16S rRNA genes with those available in GenBank [58] and the EzBioCloud server (www.ezbiocloud.net/ accessed on 8 September 2021) databases [59].

## 3. Results

### 3.1. Characteristics of the Hot Springs Studied

The numerous thermal springs of Uzon Caldera, Mutnovskii Volcano and Geyser Valley varied significantly in size and physicochemical characteristics. Most of the experiments were performed in Uzon Caldera; two samples were taken from the hot springs of the Mutnovskii Volcano area (HS01 and HS08) and only one from Geyser Valley (HS66). In total, 15 hot springs showed temperatures ranging from 52 to 90 °C, pH levels ranging from 2.5 to 6.6 and sulfate concentration ranging from 0.2 to 9.9 mM. The characteristics of sampling sites, as well as approaches used, are summarized in Table 1.

### 3.2. Rates of Sulfate Reduction

Using radioisotope analysis, we measured SRR in samples from six thermal sources and investigated the effects of various substrates on the SRP activity (Table 2). In samples HS01, HS08, HS24 and HS27 the rates definitely indicated the occurrence of sulfate reduction, while the activity of SRP was not detected in samples HS12 and HS23. The SRR was not high and varied between 2.4 nmol/cm^3^d in Solnechny Spring and 13.0 nmol/cm^3^d in the heated soil of the Oil Site (HS27). The addition of yeast extract, ethanol or hydrogen increased sulfate reduction rates in samples HS08, HS24 and HS27, while the presence of acetate or lactate increased the activity of SRP only in samples HS08 and HS24. For sample HS23 the addition of lactate made it possible to reveal the activity of the SRP not registered in native samples, which turned to be 3.1 nmol/cm^3^d. Methanol increased sulfate reduction in sample HS24 but inhibited it in all other sampling sites. All studied substrates completely inhibited the activity of SRP in sample HS01.

### 3.3. Diversity of dsrB-Carrying Prokaryotes in Kamchatka

**Figure 1:**
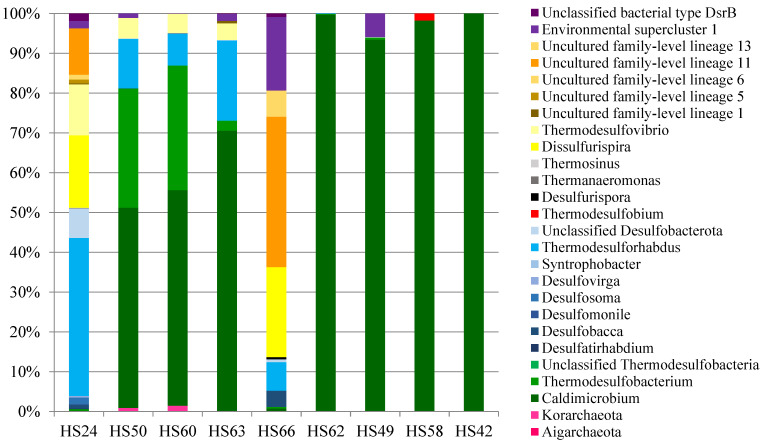
Community composition of *dsrB*-carrying microorganisms in a Kamchatka hot spring.

Communities composition of *dsrB*-carrying organisms in the Kamchatka hot springs is shown in Figure 1 (see also Figure S1 and Supplementary file 1). We were not able to isolate sufficient quantities of DNA to characterize microbial profiles in HS08, HS12 and HS27; microorganisms with reductive bacterial-type *dsrB* were not detected in samples HS01, HS17 and HS23.

The majority of *dsrB*-carrying organisms belonged to the *Desulfobacterota* phylum. They were detected in all investigated samples and comprised from 13.0 to 100.0% of all *dsrB* sequences. A significant amount of ASVs belonged to the bacteria of genus *Caldimicrobium*, thus indicating the important role of these sulfur-disproportionating (non-SRP) organisms in microbial communities of Kamchatka hot springs. Only *Caldimicrobium*-related *dsrB* sequences were detected in sample HS42, while in samples HS24 and HS66 their share were negligible (<1%). In other samples share of *dsrB* sequences belonging to *Caldimicrobium* varied from 54.2 to 99.6%.

ASVs related to *Thermodesulfobacterium* were detected in samples HS24, HS49, HS50, HS60, HS62, HS63 and HS66 with temperatures from 52 to 72 °C and with pH from 5.1 to 6.6. Their share varied from 0.2 to 31.3%. The highest share of *Thermodesulfobacterium* (30.0 and 31.3%) was found in samples HS50 and HS60 with temperatures 68 and 72 °C, respectively. In other samples with lower temperatures, representatives of the genus *Thermodesulfobacterium* were present as a minor component of the communities. For example, they comprised only 0.2 and 2.5% of all *dsrB* sequences in samples HS24 and HS63 with temperatures 52 and 55 °C, respectively. A decrease in pH also led to a decrease in the share of *Thermodesulfobacterium*, such as in samples HS62 and HS66.

ASVs related to *Thermodesulforhabdus* were present in 5 out of 12 studied communities (HS24, HS50, HS60, HS63 and HS66), where they comprised from 7.2 to 39.7% of all *dsrB* sequences. The highest share of *Thermodesulforhabdus* (39.7 and 20.1%) was found in samples HS24 and HS63, while their share decreased with increasing temperature in other samples and comprised 12.4 and 8.1% in samples HS50 and HS60. Therefore, bacteria of genus *Thermodesulforhabdus* prefer cooler habitats than the representatives of genus *Thermodesulfobacterium*. A decrease in pH also led to a decrease in the share of *Thermodesulforhabdus*, such as in samples HS62 and HS66.

ASVs related to *Desulfosoma* were present only in sample HS24 of Solnechny Spring (1.7%). Other *Desulfobacterota*-related *dsrB* sequences were assigned to mesophilic sulfate-reducing genera: *Desulfovirga*, *Syntrophobacter, Desulfatirhabdium*, *Desulfobacca* and *Desulfomonile* and were present in samples HS24 and HS66. Their share was negligible (<1%).

The next most frequent group of *dsrB* sequences was related to *Nitrospirota* lineages. ASVs belonged to *Thermodesulfovibrio* were present in samples HS24, HS50, HS60 and HS63 with temperatures of 52–72 °C and with pH 5.6–6.6, where they comprised from 4.2 to 12.8% of all *dsrB* sequences, accompanying *Thermodesulforhabdus*. ASVs related to *Dissulfurispira* were detected in samples HS24 and HS66 and comprised 18.2 and 22.6% of all *dsrB* sequences respectively.

A small amount of ASVs belonged to other lineages of *dsrB*-carrying organisms. So, ASVs belonging to *Firmicutes* lineages were found only in sample HS66, where *dsrB* sequences related to *Desulfurispora* comprised 0.6%. Sequences related to *Thermodesulfobium* (*Thermodesulfobiota* lineage) were detected in a hot spring named Arkashin Shurf (HS58) with the temperature of 64 °C and pH 5.0, where they comprised 1.7% of all *dsrB* sequences. ASVs related to *Korarchaeota* were present in samples HS50 and HS60. Their share was 0.8 and 1.5% of all sequences *dsrB* respectively.

Unassigned sequences were detected in microbial communities HS24 and HS66. Their share was 18 and 47% of all sequences *dsrB*, respectively. Phylogenetic analysis (Figure S1) indicated that unassigned *dsrB* sequences of microbial communities HS24 and HS66 were related to environmental clusters, including the Unclassified bacterial type DsrB, Environmental supercluster 1, Uncultured family-level lineages 1, 5, 6, 11 and 13 (according to Müller et al., 2015 [32] and Pelikan et al., 2016 [33]).

### 3.4. Sulfate-Reducing Prokaryotes Detected by 16S rRNA Gene-Based Analyses

**Figure 2:**
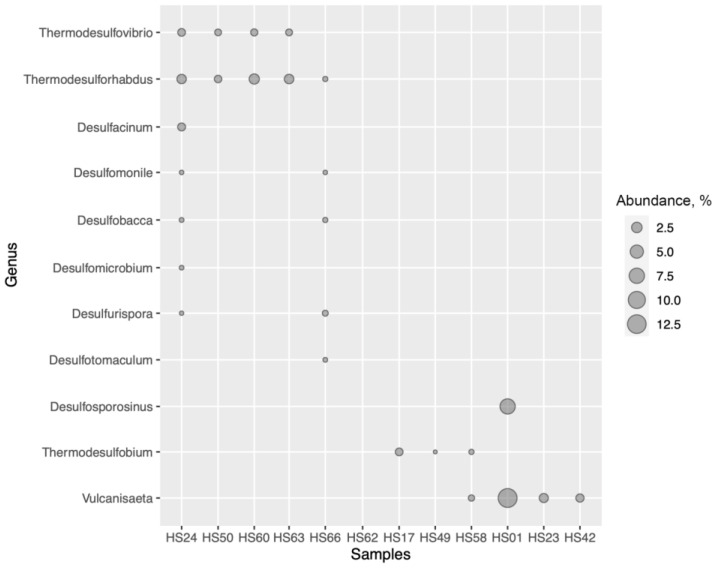
SRP detected by high-throughput sequencing of 16S rRNA gene fragments.

In order to determine the share of known SRP in microbial communities of the studied hot springs, we did the profiling of these communities by means of high-throughput sequencing of 16S rRNA gene fragments (Figure 2 and Figure S2).

Sulfate-reducing representatives of *Desulfobacterota*, *Nitrospirota* and *Firmicutes* were present in 5 out of 12 studied microbial communities (HS24, HS50, HS60, HS63 and HS66), which is in good agreement with the results of *dsrB* amplicon sequencing. However, we failed to find representatives of the genus *Thermodesulfobacterium*, whose *dsrB* sequences were detected by *dsrB* amplicon sequencing in this group of hot springs. ASVs classified as *Thermodesulforhabdus* (*Desulfobacterota*) comprised from 0.1 to 2.3% of all sequences in all five communities, while representatives of genus *Desulfacinum* (*Desulfobacterota*) were present only in sample HS24 (1.0%). We also found representatives of mesophilic sulfate-reducing *Desulfobacterota* (*Desulfomicrobium*, *Desulfobacca* and *Desulfomonile*) in microbial communities HS24 and HS66, but their share was negligible (<0.1%). The representatives of the genus *Thermodesulfovibrio* (*Nitrospirota*) were present in four microbial communities (HS24, HS50, HS60 and HS63), accompanying *Thermodesulforhabdus*. Their share varied from 0.5 to 0.9% of all sequencing of 16S rRNA gene fragments. Thermophilic sulfate-reducing representatives of the phylum *Firmicutes* were found only in spring HS66, where ASVs classified as *Desulfurispora* and *Desulfotamaculum* comprised 0.3 and 0.1% of all sequences, respectively.

Representatives of genus *Thermodesulofobium* were present in two microbial communities—HS17 and HS58, where their ASVs comprised 0.9 and 0.1% of all sequences respectively. Other representatives of sulfate-reducing bacteria could not be identified in these samples, neither by 16S rRNA, nor by *dsrB* profiling.

Recently the ability to dissimilatory sulfate reduction was proven for “*Candidatus* Vulcanisaeta moutnovskia” [5]. Representatives of genus *Vulcanisaeta* were present in four microbial communities (HS01, HS23, HS42 and HS58), where their ASVs comprised from 0.4 to 12.9% of all sequences. The highest share of *Vulcanisaeta* was detected in microbial community HS01 with temperature 90 °C and pH 3.5. The share of *Vulcanisaeta* in other hot springs was significantly lower, possibly due to temperature decrease and pH increase. It is important to note that representatives of *Vulcanisaeta* were not detected by *dsrB* amplicon sequencing because we used primer set specific for reductive bacterial-type *dsrB*, while representatives of *Crenarchaeota* phylum contain reductive archaeal-type *dsrB*. Surprisingly representatives of a mesophilic genus *Desulfosporosinus* (phylum *Firmicutes*) were also detected in microbial community HS01.

Thus, the 16S rRNA taxonomic profiling showed that known SRP constituted a minor group (0.5–3.8% of all sequences) in 9 out of 12 studied communities. Only in microbial community HS01 did the representatives of genus *Vulcanisaeta* comprise a significant group. SRP were not detected in 2 out of 12 studied communities (HS49 and HS62).

### 3.5. Cultivation of Thermophilic Sulfate-Reducing Prokaryotes

Samples HS24, HS27, HS58, HS62 and HS66 were used for the isolation of SRP (Table 3). After 4 days of incubation, an evident sulfide production accompanied by microbial growth was observed. After three subsequent transfers and consequent serial ten-fold dilutions in the same medium, only one morphological type was observed in each of the highest growth-positive dilutions. Three different strains of thermophilic SRP were isolated from the Kamchatka hot springs. Strains 3427-1 and 3458-1 reduced sulfate under chemolithoauthotrophic conditions in the presence of hydrogen. They were found to be the strains of *Thermodesulfobium acidiphilum* 3127-1^T^ that was also recovered from Kamchatka hot springs [16]. Strain 3462-1 was capable of sulfate reduction under chemolithoheterotrophic conditions in the presence of hydrogen as an electron donor and acetate as a carbon source. The phylogenetic analysis revealed that this new isolate belonged to the genus *Thermodesulfovibrio* (phylum *Nitrospirae*) and, possibly, represented a new species (Figure S3).

A stable microbial association was obtained from Solnechny Spring (HS24) that was capable of sulfate reduction under chemoorganoheterotrophic conditions in the presence of lactate as the growth substrate. Using 16S rRNA high-throughput sequencing, we showed that this association consisted of bacterial genera *Thermodesulforhabdus* (74% of all sequences of 16S rRNA gene fragments) and *Thermoanaerobacter* (23% of all sequences). Another stable microbial association was obtained from the spring HS66 in the presence of ethanol and sulfate. This association consisted of representatives of *Desulfotomaculum* (97% of all sequences) and *Thermoanaerobacterium* (2% of all sequences).

## 4. Discussion

Dissimilatory sulfate reduction plays an important role in organic matter mineralization under anoxic conditions, because SRP can use various organic compounds, usually low-molecular-mass ones, as well as hydrogen, as electron donors; all these substrates are produced at the initial stages of organic matter degradation by anaerobic microbial communities. At the same time, in volcanic habitats, molecular hydrogen can be of abiotic origin. It is utilized by lithoautotrophic thermophilic prokaryotes, including SRP, which further provide energy substrates for aerobic and anaerobic organotrophs, and, thus, act as primary producers.

In this work we compare three datasets: (a) the rates of sulfate reduction in terrestrial hot springs with different parameters and the effect of different energy substrates addition on SRP activity; (b) the information on SRP diversity in terrestrial hot springs; (c) the characteristics of SRP isolated from the same sources.

Most of the sulfate reduction rates (SRR) measurements with ^35^S-sulfate under thermophilic conditions were performed in Yellowstone National Park hot springs [60,61,62,63] and Kamchatka Peninsula [5,20,21,22,64]. SRR observed at different springs varied significantly. The highest SRR (91–37,000 nmol/cm^3^d) were observed in algal-bacterial mats of Yellowstone National Park growing at moderate temperatures (38–60 °C). It was proposed that rates were high because of the presence of readily available labile organic compounds from algal-bacterial mats or the inherent mesophilic microbial population [62]. SRR in sediments was lower and varied in the interval from 1.0 to 704 nmol/cm^3^d in hot springs of Yellowstone National Park and from 1.0 to 12.9 nmol/cm^3^d in hot springs of Kamchatka Peninsula. No correlation with sulfate concentration and pH of the spring waters was evident, but a weak negative correlation existed between SRR and temperature [62]. It is important to note that replicate samples showed a large deviation in the measured SRR both for algal-bacterial mats and for sediments [21,62]. For example, the maximum value of SRR in sediment (704 nmol/cm^3^d) was determined only in one core of Norris Geyser Basin (Site C); the other two cores gave results below the detection limit [60]. Our estimates of SRR (2.4–13.0 nmol/cm^3^d) in hot springs of Uzon Caldera agree well with the previous data on the activity of this process in hot springs sediments.

The addition of energy substrates increased microbial activity in substrate-limited systems. At Yellowstone National Park no SRR increase was observed during formate-, acetate- and lactate-supplied incubation experiments. Furthermore, in certain cases, SRR decreased in comparison with the in situ rates [62]. In this study, we observed that substrates addition could either increase or decrease SRR in Uzon Caldera hot springs isolated samples.

The addition of any of the studied substrates (hydrogen, methanol, ethanol, acetate, lactate or yeast extract) led to an increase in SRR in the sample of Solnechny Spring (HS24). *drsB* profiling of microbial community of Solnechny Spring revealed that dominating their SRP were related to *Thermodesulforhabdus* (39.7%) and *Thermodesulfovibrio* (12.8%). *Thermodesulforhabdus norvegica* isolated from the North Sea oil deposit is the only cultivated neutrophilic and moderate thermophilic representative of the genus, capable of sulfate reduction using a wide range of organic compounds, including ethanol, acetate, lactate [9]. *drsAB* and/or 16S rRNA analysis revealed representatives of this genus in oil reservoirs and wells of Japan and China, Cu-Pb-Zn underground mine, Guaymas Basin in the Gulf of California, and hot spring of Yellowstone National Park [65,66,67,68,69]. Cultivated *Thermodesulfovibrio* are also moderately thermophilic and neutrophilic sulfate-reducing bacteria isolated both from natural thermal habitats (terrestrial hot springs) and anthropogenic ones (methanogenic sludge). In the presence of sulfate, *Thermodesulfovibrio* spp. grow on hydrogen (with acetate as carbon source), lactate, and pyruvate [8,70,71,72]. In Solnechny Spring, ASVs related to *Desulfosoma* and *Thermodesulfobacterium* comprised 1.7% and 0.4% of all *dsrB* sequences, respectively. Two known species of genus *Desulfosoma* isolated from terrestrial hot springs are moderate thermophiles living at neutral pH and growing heterotrophically on diverse organic substrates in the presence of sulfate as the terminal electron acceptor. *Desulfosoma caldarium* is also capable of autotrophic growth in the presence of hydrogen [73,74]. Cultivated *Thermodesulfobacterium* are thermophilic and neutrophilic sulfate-reducing bacteria that were isolated from natural ecosystems such as terrestrial hot springs, deep-sea hydrothermal vents, high-temperature oil reservoirs. In the presence of sulfate, representatives of the genus *Thermodesulfobacterium* use hydrogen as the electron donor; some species also can use ethanol, acetate, lactate and pyruvate [6,7,71,75,76].

Thus, the growth parameters of *Thermodesulforhabdus norvegica*, as well as of cultivated species of *Thermodesulfovibrio*, *Desulfosoma* and *Thermodesulfobacterium* correspond to temperature and pH of Solnechny Spring, while the range of growth substrates used by these microorganisms explains the increase in SRR at addition of hydrogen, methanol, ethanol, acetate, lactate.

The share of *dsrB* sequences related to mesophilic sulfate-reducing lineages of *Desulfobacterota* phylum (*Desulfovirga*, *Syntrophobacter*, *Desulfatirhabdium*, *Desulfobacca* and *Desulfomonile*) was negligible (<1%). The presence of mesophilic sulfate-reducing lineages of *Desulfobacterota* in the hot springs may indicate either the presence of new thermophilic/thermotolerant species in these genera or a possible drift from surrounding cooler areas.

The 16S rRNA taxonomic profiling of the Solnechny Spring microbial community showed the presence of bacteria belonging to *Thermodesulforhabdus*, *Thermodesulfovibrio*, *Desulfacinum*, *Desulfomicrobium*, *Desulfobacca* and *Desulfomonile*, and, thus, is in good agreement with *dsrB* amplicon sequencing results. However, we failed to find representatives of genus *Thermodesulfobacterium*, whose *dsrB* sequences were detected by *dsrB* amplicon sequencing; instead, representatives of genus *Desulfacinum* were detected that were not revealed by *dsrB* genes analysis. Currently, three species are described in the genus *Desulfacinum*, which were isolated from high-temperature oil reservoirs, shallow-water submarine thermal environments or terrestrial hot springs [77,78,79,80]. It should be noted that bacteria of the genus *Desulfacinum* are closely related to those of genus *Desulfosoma*, whose *dsrB* sequences were detected in this hot spring. Thus, maybe both 16S rRNA and *dsrB* profiling data indicate the same microorganism.

The 16S rRNA taxonomic profiling showed that sulfate-reducing bacteria mentioned above constituted a minor group in the community on Solnechny Spring (3.8% of all sequences). Nevertheless, we managed to get a stable microbial association capable of sulfate reduction in the presence of lactate. The main component of this microbial association was *Thermodesulforhabdus* sp. Moreover, a thermophilic microbial consortium performing anaerobic autotrophic oxidation of hydrothermal siderite was recently obtained from Solnechny Spring [81]. Representatives of genus *Thermodesulfovibrio* played an important role in this consortium. The microbial community of Solnechny Spring was also studied by Menzel and colleagues using metagenomic analyses, but the diversity of SRP was not discussed [29].

In general, the results of *dsrB* and 16S rRNA genes profiling are in agreement with the physical and chemical parameters of the hot springs studied in this work, dividing them into several groups. The most numerous one includes Solnechny Spring and other neutrophilic or slightly acidic hot springs (HS50, HS60, HS62, HS63 and HS66) with moderate temperatures. These springs also showed the presence of *dsrB*-carrying microorganisms belonging to sulfate-reducing lineages of *Desulfobacterota* (*Thermodesulforhabdus*, *Thermodesulfobacterium* and *Desulfobacca*), *Nitrospirota* (*Thermodesulfovibrio*) and *Firmicutes* (*Desulfurispora*). With the temperature increase, the share of *Thermodesulforhabdus* and *Thermodesulfovibrio* decreased, while the share of *Thermodesulfobacterium* increased. These results are in agreement with the growth temperature ranges of the abovementioned bacteria. In the hot spring HS66, ASVs related to *Desulfurispora* were detected. *Desulfurispora thermophila*, the only described species of this genus, was isolated from a sulfidogenic fluidized-bed reactor treating acidic metal- and sulfate-containing water [10]. The reported temperature and pH range for growth of *Desulfurispora* correspond to temperature and pH in HS66 hot spring. Our work is the first to reveal the presence of *Desulfurispora* spp. in terrestrial hot springs.

The 16S rRNA taxonomic profiling confirmed the presence of bacteria of genera *Thermodesulforhabdus*, *Desulfobacca*, *Thermodesulfovibrio* and *Desulfurispora* in microbial communities of hot springs HS50, HS60, HS62, HS63 and HS66, but, same as in the case with Solnechny Spring, we failed to find representatives of genus *Thermodesulfobacterium*. That indicates the advantages of using two approaches and revealing the diversity in a certain group of prokaryotes both by phylogenetic and functional markers. The share of known cultured SRP in microbial communities HS50, HS60, HS62, HS63 and HS66 comprised up to 3.0% of all sequences. The 16S rRNA profiling also showed the presence of bacteria of genus *Desulfotamaculum* in microbial community HS66. Representatives of this genus are moderate thermophiles and are commonly found in deep subsurface environments, such as mines [82,83,84], aquifers [82,85,86], hot oil-field waters [87] and hydrothermal vents sediments [88,89]. The reported temperature range of *Desulfotamaculum* corresponds to the temperature in the hot spring HS66.

The stable microbial association obtained from the hot spring HS66 was capable of sulfate reduction using ethanol as a growth substrate and contained *Desulfotomaculum* as the main component. From the sample of spring HS62, strain 3462-1 was isolated. The phylogenetic analysis revealed that the new isolate belonged to the genus *Thermodesulfovibrio* (phylum *Nitrospirae*) where it, possibly, represented a new species.

Similar to Solnechny Spring, the addition of hydrogen, ethanol, acetate, lactate or yeast extract led to an SRR increase in the spring HS08 sample. We were not able to isolate DNA from this sample in amounts sufficient for *dsrB* and 16S rRNA genes profiling but succeeded in strain 3408-1 isolation. Recently this strain, assigned to the phylum *Firmicutes*, was described as *Desulfothermobacter acidiphilus* gen nov., sp. nov. [17]. It is a moderately thermoacidophilic anaerobe able to grow either by sulfate or thiosulfate respiration with H_2_ or formate as substrates or by fermentation of yeast extract, maltose, sucrose, glucose and pyruvate. These data are in agreement with the increase in SRR caused by hydrogen addition. Other substrates can increase SRR indirectly, or by stimulating the activity of other sulfate-reducing bacteria present in this habitat.

The second group of springs includes moderately acidic hot springs HS17, HS27 (Oil Site) and HS58 (Arkashin Shurf), where representatives of the genus *Thermodesulofobium* were detected. Recent phylogenetic studies showed that this genus represents a distinct phylum-level lineage *Thermdesulvobiota* [90,91,92,93]. *Thermodesulfobium narugense* and *Thermodesulfobium acidiphilum* are thermoacidophilic autotrophs coupling the oxidation of hydrogen or formate with sulfate or thiosulfate reduction [16,94]. Growth parameters of cultivated *Thermodesulfobium* spp. corresponded to temperature and pH values of the hot springs HS17, HS27 and HS58. Our estimates of SRR in the heated soil of the Oil Site (HS27) showed that sulfate respiration increased at the addition of hydrogen and yeast extract, while lactate, acetate and ethanol did not produce any significant impact. Methanol completely inhibited the activity of SRP. We assume that hydrogen is oxidized directly by representatives of genus *Thermodesulfobium*, while the yeast extract increased SRR indirectly, being degraded with hydrogen formation by fermentative bacteria present in the sample. drsB and 16S rRNA gene fragments profiling showed that *Thermodesulfobium* constituted a minor group in this pool of springs. We succeeded in the isolation of two new strains of *Thermodesulfobium* from a sample of Oil Site heated soil (HS27) and Arkashin Shurf hot spring (HS58). Representatives of genus *Thermodesulfobium* were detected in these springs before [16,25].

Previously it was assumed that representatives of family *Thermoproteaceae* assigned to the phylum *Crenarchaeota* may also be capable of growth by means of sulfate respiration [16,22,95,96,97]. Recently this process was proved for “*Candidatus* Vulcanisaeta moutnovskia”, but not for other hyperthermophilic *Crenarchaeota* [5]. In the course of this work representatives of genus *Vulcanisaeta* were detected in hot springs HS01, HS23, HS42 and HS58 in the course of 16S rRNA gene fragment profiling. The largest share of *Vulcanisaeta* (12.9%) was determined in microbial community HS01 with extremely thermoacidophilic conditions (90 °C; pH 3.5). Other springs had lower temperatures or higher pH, and the share of *Vulcanisaeta* was significantly lower. It is worth noticing that representatives of *Vulcanisaeta* were not detected by *dsrB* amplicon sequencing, as the primer set used was specific for reductive bacterial-type *dsrB*, while representatives of Crenarchaeota phylum contain reductive archaeal-type *dsrB*. However, SRR measurement showed a prominent sulfate reduction process that took place in HS01 hot spring. Our results repeat previously published data according to which representatives of the genus *Vulcanisaeta* are responsible for the dissimilatory sulfate reduction in Oreshek Spring [5]. Thus, we can assume that in hot springs HS01, HS23, HS42 and HS58 *Vulcanisaeta* is responsible for the dissimilatory sulfate reduction.

Interestingly, at substrate addition, the SRR decreased in comparison with the in situ rates. The decrease in in situ sulfate reduction in the presence of different electron donors could be explained by the activation of other groups of microorganisms competing for nutrients in this harsh environment.

Surprisingly, representatives of genus *Desulfosporosinus* (*Firmicutes*) were detected in microbial community HS01 by 16S rRNA gene fragments profiling, while no microorganisms with reductive bacterial-type *dsrB* were detected in this spring. All currently known cultivated *Desulfosporosinus* are mesophilic endospore-forming sulfate-reducing bacteria that do not grow at temperatures above 40 °C, but, at the same time, the only mesophilic sulfate reducers that perform sulfate reduction in acidic conditions [2]. The presence of representatives of the genus *Desulfosporosinus* in the HS01 microbial community may indicate either a possible drift from surrounding cooler acidic areas or the presence of new thermophilic non-sulfate-reducing species in this genus.

For the correct interpretation of obtained environmental *dsrB* diversity data, it is worth considering that reductive *dsrB* genes are present not only in SRP but also in sulfite-reducing and sulfur-disproportionating prokaryotes, as well as in microorganisms that produce sulfite intercellular by degrading organosulfonates and those that apparently have lost the ability of respiration with sulfur compounds [32]. There is still no way to discriminate between SRP and other groups of *dsrB*-carrying organisms [98]. In this work, it was shown that a significant amount of *dsrB* ASVs belonged to the bacteria of genera *Caldimicrobium* and *Dissulfurispira*. Bacteria of genus *Caldimicrobium* is a widely distributed group of sulfur-metabolizing lithoautotrophs inhabiting Kamchatka hot springs [14]. However, representatives of both genera are sulfur-disproportionating bacteria that are not capable of dissimilatory sulfate reduction [99,100,101]. Therefore, *Caldimicrobium* and *Dissulfurispira* were not regarded here as the representatives of SRP. The same might concern the representatives of the phylum *Korarchaeota* detected in microbial communities HS50 and HS60, as recently it was suggested that “*Candidatus* Methanodesulfokores washburnensis” may perform sulfite or thiosulfate reduction, but not sulfate reduction [102].

It is not possible to decide if uncultured microorganisms possessing *dsrB* genes are sulfate reducers, or belong to other groups of sulfur-metabolizing prokaryotes. Still, their presence in Kamchatka hot springs could become a driver for further investigations. In this view, Solnechny Spring (HS24) and spring HS66 located in Geyser Valley are of special interest. In Solnechny Spring uncultured microorganisms of family-level Lineage 11 [32] comprised 11.7% of all *dsrB*-bearing population. Spring HS66 was unique in terms of the diversity of *dsrB*-possessing uncultured prokaryotes, as, in addition to Lineage 11 (37.8% of all *dsrB* sequences), it contained also the representatives of Environmental supercluster 1 (18.5%) and of Lineage 13 (6.6%). Lineage 11 belongs to *Desulfobacterota* supercluster, lineage 13—to *Nitrospirota* supercluster, while, so far, thermophiles have not been registered in both these groups. Environmental supercluster 1, among others, includes also *dsrB* sequences obtained from thermal environments [32] Thus, these data expand the knowledge on uncultured thermophilic prokaryotes participating in sulfate reduction, or other sulfur-metabolizing processes in thermal environments.

Summarizing the data presented in this work, we can conclude that dissimilatory sulfate reduction takes place in sediments of Kamchatka hot springs with different physical and chemical parameters, but sulfate reduction rates are comparatively low and SRP constituted minor groups in most of the studied communities. At the same time, using different approaches we registered a significant diversity of sulfate-reducing prokaryotes, and according to the composition of their communities, three groups of hot springs were revealed. The first and most numerous group was that of moderately thermophilic neutral or slightly acidic springs with a wide functional diversity of thermophilic sulfate reducers able to use a wide range of electron donors. Two other groups comprised acidic hot springs, either with moderate temperatures inhabited by *Thermodesulfobium*, or extremely hot ones populated by archaea of genus *Vulcanisaeta*. While *Thermodesulfobium* is a lithoautotroph utilizing molecular hydrogen as an electron donor for sulfate reduction, the growth substrate of *Vulcanisaeta* remains unknown.

In the course of samples incubation in the presence of ^35^S-labeled sulfate indicated that the sulfate reduction process is limited by the availability of electron donors. Most probably, sulfate reducers fail to outcompete other thermophilic prokaryotes performing anaerobic respiration. For example, the bacteria of genus *Caldimicrobium* were dominating among *dsrB*-possessing microorganisms in seven of nine springs studied, evidently winning the competition for molecular hydrogen. It could be explained either by the higher availability of elemental sulfur in comparison with sulfate or by a higher affinity of *Caldimicrobium* to the substrate.

Thus, our work demonstrated a wide diversity of SRP inhabiting Kamchatka hot springs. Our results also show that the rate of sulfate reduction is limited by the availability of electron donors and by the competition with other anaerobic bacteria. It does not exclude the existence of thermophilic microbial communities where sulfate reduction achieves higher rates and plays an important role in sulfur and carbon cycles in these habitats. Of special interest is the role of well-represented uncultured *dsrB*-possessing bacteria found in diverse thermal springs of Kamchatka.

## 5. Conclusions

Thus, radioisotopic experiments with ^35^S-labeled sulfate showed that microbial communities of the Kamchatka hot springs are actively reducing sulfate. However, SRP constituted minor groups in most of the studied communities. Apparently, SRP functions at the final stages of organic matter degradation by anaerobic microbial communities of the Kamchatka hot springs and compete for substrates with other physiological groups of anaerobic organisms. SRP belonging to *Desulfobacterota* (*Thermodesulfobacterium*, *Thermodesulforhabdus*, *Desulfosoma*/*Desulfacinum*), *Nitrospirota* (*Thermodesulfovibrio*) and *Firmicutes* (*Desulfurispora*, *Desulfotamaculum*, *Desulfothermobacter*) were found to inhabit neutrophilic and slightly acidic hot springs with a pH from 5.1 to 6.6 and temperatures from 52 to 72 °C, while bacteria belonging to *Thermodesulofibiota* (*Thermodesulofibium*) prefer moderate acidic hot springs with a pH from 4.2 to 5.0 and temperatures from 53 to 65 °C. The microbial community HS01 represents an extremely thermoacidophilic environment with a temperature of 90 °C and a pH of 3.5, where sulfate reduction is mediated by archaea of the phylum *Crenarchaeota*, the most likely candidates being chemoorganoheterotrophic strains of genus *Vulcanisaeta*. Moreover, the presence of the Unclassified bacterial type DsrB, Environmental supercluster 1, Uncultured family-level lineages 1, 5, 6, 11 and 13 illustrates that there is still unexplored diversity of *dsrAB*-containing microorganisms in the hot springs of Kamchatka and further studies need to be performed.

## Figures and Tables

**Table 1:** Main characteristics of studied Kamchatka hot springs.

Sample Name	Spring ^1^, Coordinates	Sample Description	T (°C)	pH	SO_4_^2−^, mM	Approach Applied ^2^
Foot of Mutnovskii volcano:						
HS01	Unnamed; 52°31.809′ N 158°11.472′ E, 823 m	Yellow to gray deposit	90	3.5	3.2	HTS, RT
HS08	Unnamed; 52°32.087′ N 158°11.851′ E, 794 m	Black deposit	60	5.6	1.1	RT
Uzon Caldera:						
HS12	Unnamed, OTF; 54°30.413′ N 160°00.043′ E, 659 m	Gray deposit	82	2.5	9.9	RT
HS17	Unnamed, CTF; 54°30.008′ N 160°00.322′ E, 657 m	Yellow/orange deposit	53	5.0	1.2	HTS
HS23	Unnamed, WTF; 54°30.009′ N 159°56.983′ E, 707 m	Gray deposit	72	5.0	2.7	HTS, RT
HS24	Solnechny, CTF; 54°29.941′ N 159°59.530′ E, 657 m	Black deposit	52	6.1	0.6	HTS, I, RT
HS27	Oil Site, CTF; 54°30.023′ N 160°00.088′ E, 654 m	Black deposit	61	4.2	2.2	RT, I
HS42	Sery, ETF; 54°29.882′ N 160°00.862′ E, 662 m	Short gray filaments around the margins	80	6.1	1.9	HTS
HS49	Unnamed, ETF; 54°29.892′ N 160°00.870′ E, 664 m	Short white filaments around the margins	63	6.3	0.5	HTS
HS50	Unnamed, ETF; 54°29.892′ N 160°00.870′ E, 664 m	Short gray filaments around the margins	72	6.6	0.2	HTS
HS58	Arkashin Shurf, CTF; 54°30.000′ N 160°00.337′ E, 660 m	Yellow/orange deposit	64	5.0	0.8	HTS, I
HS60	Unnamed, CTF; 54°30.013′ N 160°00.441′ E, 657 m	White filaments	68	6.1	1.7	HTS
HS62	Unnamed, ETF; 54°29.993′ N 160°00.810′ E, 659 m	Gray deposit	72	5.1	1.5	HTS, I
HS63	Vertoletny, ETF; 54°30.005′ N 160°00.732′ E, 664 m	Gray deposit	55	5.6	0.6	HTS
Valley of Geysers:						
HS66	Unnamed; 54°26.299′ N 160°08.375′ E, 451 m	Black deposit	62	5.3	1.2	HTS, I

^1^ WTF west thermal field, ETF east thermal field, CTF central thermal field, OTF orange thermal field (Uzon Caldera). ^2^ RT radioisotopic tracing, HTS high-throughput sequencing of 16S rRNA and *dsrAB* genes fragments, I isolation.

**Table 2:** Sulfate reduction rates observed in microcosm experiments amended different substrates.

Substrate	Sulfate Reduction Rates (nmol/cm^3^d) ^1^					
	HS24	HS08	HS27	HS01	HS23	HS12
In situ	2.4 ± 0.5	3.1 ± 1.2	13.0 ± 3.6	8.7 ± 3.0	ND	ND
Hydrogen	24.2 ± 7.6	34.2 ± 4.4	136.6 ± 2.2	ND	ND	ND
Acetate	33.5 ± 1.5	5.9 ± 0.1	7.0 ± 3.4	ND	ND	ND
Lactate	20.5 ± 0.6	18.2 ± 1.2	12.9 ± 4.8	ND	6.2 ± 3.1	ND
Ethanol	41.7 ± 3.8	83.4 ± 8.1	19.9 ± 0.1	ND	ND	ND
Methanol	8.7 ± 0.9	2.4 ± 0.4	ND ^2^	ND	ND	ND
Yeast extract	45.6 ± 8.2	25.3 ± 4.8	98.3 ± 8.4	ND	ND	ND

^1^ Activities were measured in nmol of SO_4_^2−^ consumption per cm^3^ of sediment per day (nmol/cm^3^d). ^2^ ND not detected.

**Table 3:** SRP isolated from Kamchatka hot springs.

Sample	Isolate	Growth, T °C	Growth, pH	Electron Donor	Carbon Source	Closest Relative	16S rRNA Gene Identity, %
HS62	3462-1	65	5.5	H_2_	Acetate	*Thermodesulfovibrio aggregans*	97.6
HS27	3427-1	55	4.7	H_2_	CO_2_	*Thermodesulfobium acidiphilum*	99.9
HS58	3458-1	55	5.0	H_2_	CO_2_	*Thermodesulfobium acidiphilum*	99.9
HS24	Microbial association	50	6.0	Lactate	Lactate	*Thermodesulforhabdus* (74%) ^1^ *Thermoanaerobacter* (23%) ^1^	96.0 ^2^ 100.0 ^2^
HS66	Microbial association	60	5.0	Ethanol	Ethanol	*Desulfotomaculum* (97%) ^1^ *Thermoanaerobacterium* (2%) ^1^	98.0 ^2^ 99.0 ^2^

^1^ The values in brackets indicate share of microorganism in the microbial association. ^2^ Only for V3–V4 region of the 16S rRNA gene.

## Data Availability

All the sequencing data are deposited in NCBI BioProject PRJNA753547.

## Supplementary Materials

The following are available online at https://www.mdpi.com/article/10.3390/microorganisms9102072/s1. Figure S1: Phylogeny of *dsrB* amplicons, Figure S2: Relative abundance of phyla of prokaryotes in the studied microbial communities, Figure S3: A 16S rRNA gene sequence-based maximum-likelihood phylogenetic tree, showing the position of strain 3462-1. Supplementary file 1: results of *dsrB* genes amplification, sequencing and analyses.
