# Supplementary material for: Diversity and Activity of Sulfate-Reducing Prokaryotes in Kamchatka Hot Springs
Source: Microorganisms. 2021 Oct 1;9(10):2072. doi: 10.3390/microorganisms9102072 (PMC8539903; doi:10.3390/microorganisms9102072)
Supplement: Supplementary file 1 [file microorganisms-09-02072-s001.zip › Supplementary material (Frolov et al).pdf]

Evgenii N. Frolov, Alexandra V. Gololobova, Alexandra A. Klyukina, Elizaveta A. Bonch-Osmolovskaya, Nikolay V. Pimenov, Nikolay A. Chernyh, Alexander Y. Merkel

Diversity and activity of sulfate-reducing prokaryotes in Kamchatka hot springs.

Supplementary Materials

Figure S1 Phylogeny of *dsrB* amplicons (on next page). Tree was constructed using PhyML 3.0 [103], Smart Model Selection v1.8.1 [104], Approximate Likelihood-Ratio Test for Branches [105] and previously published database [33]. Bootstrap values are shown at the nodes. Bar, 0.10 changes per position.

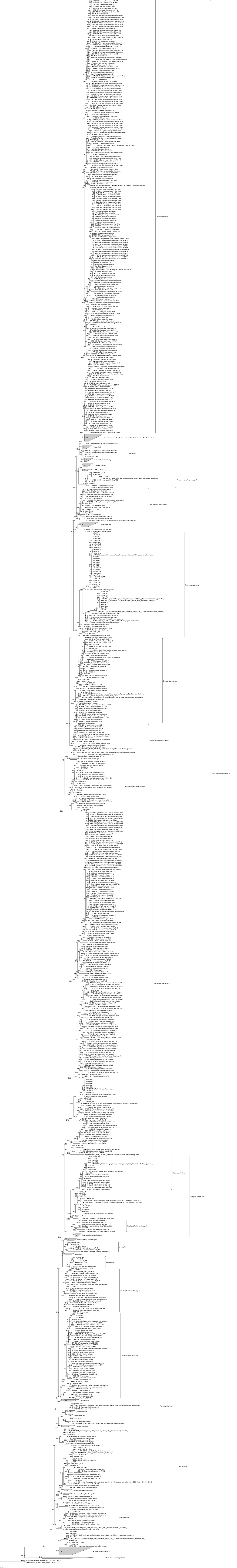

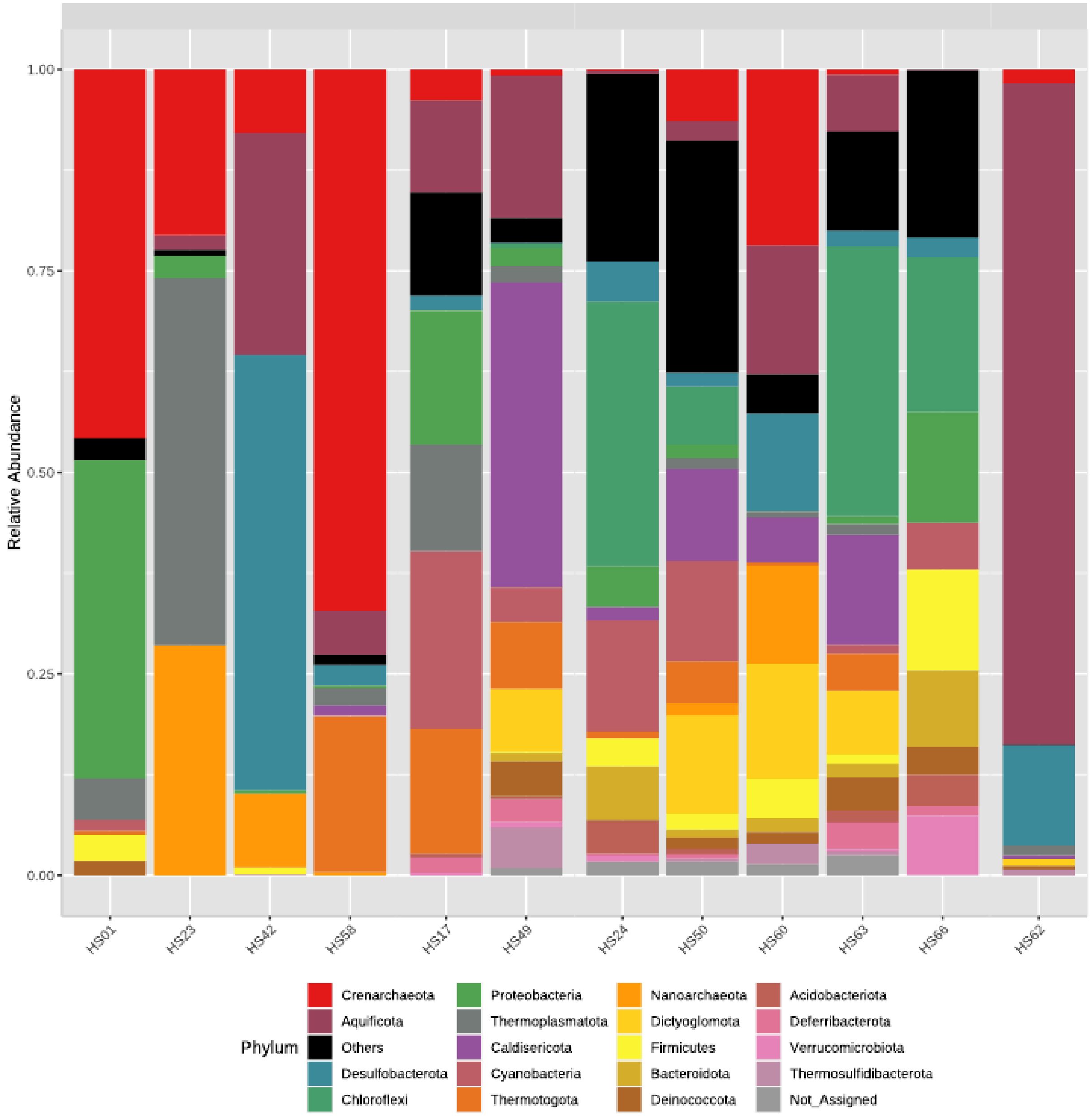

Figure S2 Relative abundance of phylums of prokaryotes in the studied microbial communities.

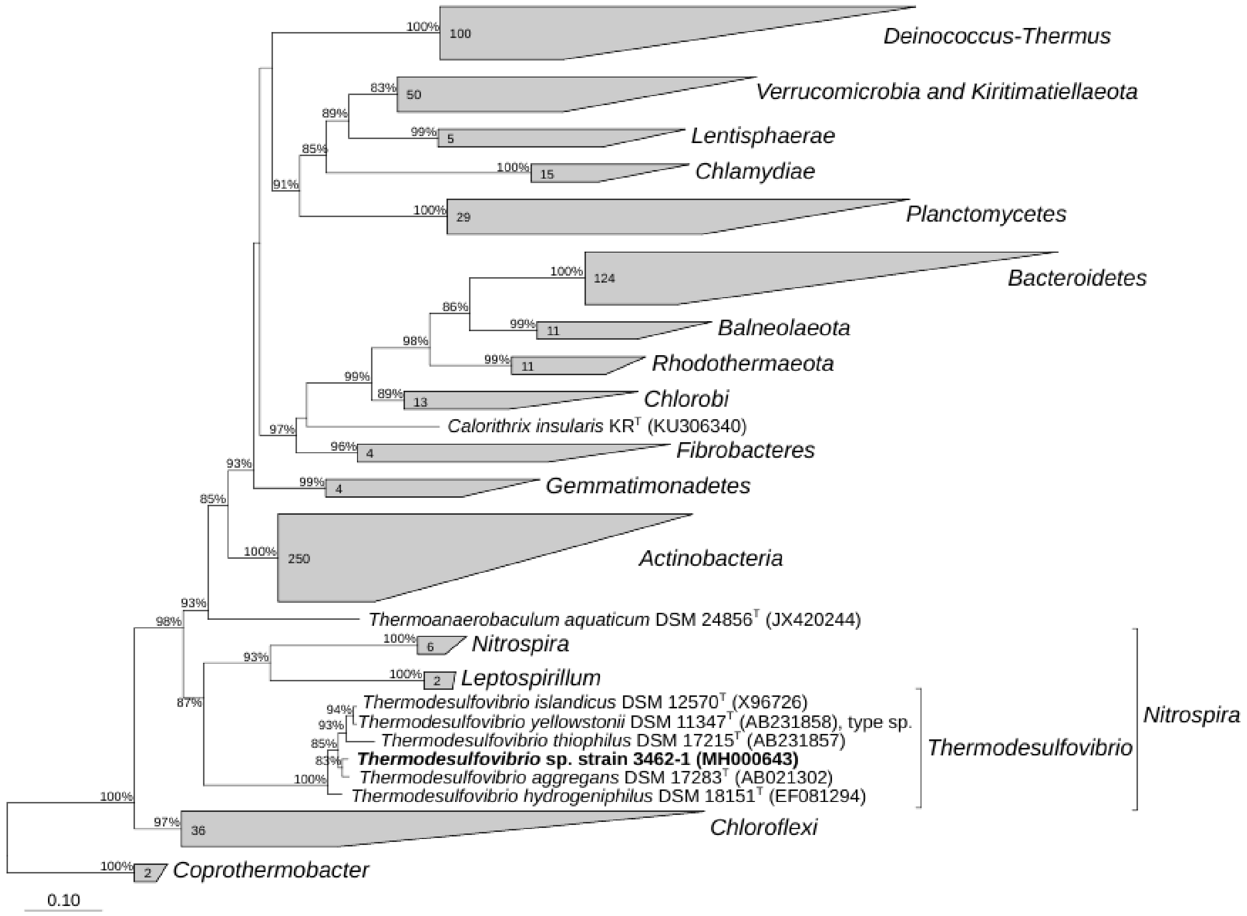

Figure S3 A 16S rRNA gene sequence-based maximum-likelihood phylogenetic tree, showing the position of strain 3462-1.

References

103. Guindon, S.; Dufayard, J.F.; Lefort, V.; Anisimova, M.; Hordijk, W.; Gascuel, O. New algorithms and methods to estimate maximum-likelihood phylogenies: assessing the performance of PhyML 3.0. *Syst. Biol.* **2010**, *59*, 307-321; DOI:10.1093/sysbio/syq010.
104. Lefort, V.; Longueville, J.E.; Gascuel, O. SMS: Smart Model Selection in PhyML. *Mol. Biol. Evol.* **2017**, *34*, 2422-2424; DOI:10.1093/molbev/msx149.
105. Anisimova, M.; Gascuel, O. Approximate likelihood-ratio test for branches: A fast, accurate, and powerful alternative. *Syst. Biol.* **2006**, *55*, 539-552; DOI:10.1080/10635150600755453.
